# Ultrasensitive Gas Refractometer Using Capillary-Based Mach–Zehnder Interferometer

**DOI:** 10.3390/s20041191

**Published:** 2020-02-21

**Authors:** Haijin Chen, Xuehao Hu, Meifan He, Pengfei Ren, Chao Zhang, Hang Qu

**Affiliations:** 1 Research Center for Advanced Optics and Photoelectronics, Department of Physics, College of Science, Shantou University, Shantou 515063, Guangdong, China; 18hjchen1@stu.edu.cn (H.C.); xhhu3@stu.edu.cn (X.H.); 17mfhe@stu.edu.cn (M.H.); 17pfren@stu.edu.cn (P.R.); 2 Key Laboratory of Intelligent Manufacturing Technology of MOE, Shantou University, Shantou 515063, Guangdong, China; 3 College of Mechanical Engineering, Yangzhou University, Yangzhou 225127, China; zhangc@yzu.edu.cn

**Keywords:** fiber-optic sensor, capillary sensor, gas refractometer, gaseous refractive index sensing, air pressure sensor

## Abstract

In this paper, we report a capillary-based Mach–Zehnder (M–Z) interferometer that could be used for precise detection of variations in refractive indices of gaseous samples. The sensing mechanism is quite straightforward. Cladding and core modes of a capillary are simultaneously excited by coupling coherent laser beams to the capillary cladding and core, respectively. An interferogram would be generated as the light transmitted from the core interferes with the light transmitted from the cladding. Variations in the refractive index of the air filling the core lead to variations in the phase difference between the core and cladding modes, thus shifting the interference fringes. Using a photodiode together with a narrow slit, we could interrogate the fringe shifts. The resolution of the sensor was found to be ~5.7 × 10^−8^ RIU (refractive index unit), which is comparable to the highest resolution obtained by other interferometric sensors reported in previous studies. Finally, we also analyze the temperature cross sensitivity of the sensor. The main goal of this paper is to demonstrate that the ultra-sensitive sensing of gas refractive index could be realized by simply using a single capillary fiber rather than some complex fiber-optic devices such as photonic crystal fibers or other fiber-optic devices fabricated via tricky fiber processing techniques. This capillary sensor, while featuring an ultrahigh resolution, has many other advantages such as simple structure, ease of fabrication, straightforward sensing principle, and low cost.

## 1. Introduction

Recently, research and development of fiber-optic gas refractometers has drawn tremendous attention due to its potential applications in both scientific and industrial fields, such as gas composition detection, gaseous lasers, environmental protection, homeland security, and atmosphere pressure monitoring [1,2]. 

Generally, variations in gas refractive index induced by pressure or temperature changes are in a narrow range of 10^−6^ to 10^−4^ refractive index unit (RIU). Thus, the majority of fiber-optic gas refractometers typically adopt interferometric sensing schemes due to their relatively high sensitivities. In the last decade, a number of Fabry–Pérot (F-P) or Mach–Zehnder (M-Z) fiber-optic sensors have been proposed and experimentally demonstrated for sensing of gas refractive index. Methods for fabricating F-P fiber sensors may include mechanically immobilizing two single-mode fibers (SMFs) in a metallic sleeve while leaving a F-P cavity between the end facets of the two fibers [3], splicing optical capillaries between two SMFs [4,5], splicing hollow-core photonic crystal fibers (HC-PCFs) between two SMFs [6,7], splicing a short piece of SMF between two SMFs with a lateral offset [8], lateral laser-milling an open F-P cavity in the core of a SMF or a multimode fiber [9], creating an air cavity between two fibers via fusion splicing [10], or cascading a SMF with two capillaries (or HC-PCFs) of different inner diameters [11,12,13]. Spectral interrogations are then performed to correlate with the changes in the refractive index with spectral shifts in reflection spectra. Sensitivities are typically found to be on the order of 10^3^–10^4^ nm/RIU. In particular, M. Quan et al. demonstrated a F-P fiber sensor fabricated by cascading a PCF to a capillary fiber fusion-spliced with a section of SMF [12]. This sensor operated based on the Vernier effect and reported an extremely high sensitivity of ~30,899 nm/RIU. Assuming that a 1-pm spectral shift could be differentiated (typical resolution of a commercial optical spectrum analyzer), this sensitivity is equivalent to a detection resolution of ~3 × 10^−8^ RIU. Though the above-mentioned F-P fiber sensors feature relatively high sensitivities, their fabrications could be technically challenging and costly. Precise fs-laser milling and tricky fiber splicing with meticulous position manipulation may be required, which not only increased complexity of the fabrication process, but also might compromise the robustness of the sensor structure. The use of PCFs and state-of-the-art spectrum analyzers also increased the expense. 

M-Z interferometric fiber sensors have also been demonstrated [14,15,16,17,18]. For example, I. Shavrin et al. designed an M-Z interferometer that used a section of SMF as one arm and a section of HC-PCF as the other [16]. Coherent light output from the two fibers generated interferograms captured by a CCD camera. Gaseous samples entering the HC-PCF modified the effective refractive index of the guided mode, thus shifting the interference fringes. Fast Fourier transform (FFT) was applied to the captured interferograms to analyze the amplitude and phase information associated with the complex refractive indices of gaseous analytes. Finally, a resolution of 10^−7^ RIU was reported. Besides, the F-P sensor proposed in [8] could also be used in a M-Z sensing scheme. By sandwiching a SMF between two SMFs with a lateral offset, the in-coupled light of the lead-in SMF would propagate via the open cavity and the partially-spliced SMF simultaneously, and eventually interfered in the lead-out SMF. Spectral interrogation of the sensor suggested a sensitivity of ~3402 nm/RIU [14]. X. Huang et al. demonstrated a fiber-optic M-Z interferometer by splicing a thin-core fiber (TCF) between two SMFs [18]. The in-coupled light from the lead-in SMF would excite both cladding modes and core modes in the TCF. Transmitted lights from the cladding and the core recombined in the lead-out SMF and thus produced modal interference in the transmission spectrum. Sensing layers consisting of poly(acrylic acid) (PAA), poly(allyamine hydrochloride) (PAH), and carboxyl-modified single-walled carbon nanotubes (SWCNTs-COOH) are deposited on the cladding of the TCF to specifically absorb ammonia (NH_3_) gas molecules. Variations in concentrations of NH_3_ lead to changes in the effective refractive index of the cladding mode guided by the TCF, thus resulting in shifts of the transmission spectra. Sensitivity of this sensor was reported to be ~31 pm/ppm (part per million) for NH_3_ detection. Note that these sensors also used expensive PCFs or required rather complicated fiber-splicing processes that brought challenges to the sensor fabrication. Moreover, if the sensor targets for detection of specific gaseous samples, the transducing area of the fiber sensor should be functionalized with certain sensing layers.

Several other fiber-optic gas refractometers utilized in-fiber resonant structures such as fiber gratings (including tilt fiber Bragg gratings or long period gratings) and surface plasmonic resonance (SPR) structures [19,20,21,22]. In these sensors, variations in refractive indices at the vicinities of the in-fiber resonant structures would modify the signature wavelength in the transmission or reflection spectra. Sensitivities of these sensors were on the order of 10^3^ nm/RIU, comparable to the interferometric fiber sensors. However, note that creating the resonant structures into fibers generally involved sophisticated phase-mask lithographic techniques and/or multiple depositions of nanometer-thick noble-metal layers.

In this paper, we propose and experimentally demonstrate an ultrasensitive gas refractometer using an optical capillary-based M-Z interferometer. A coherent laser is split into two virtually-parallel beams that are coupled to the hollow core and cladding of a capillary, exciting core modes and cladding modes thereof. The transmitted light from the cladding and the core of the capillary partially overlaps, generating an interferogram that is then captured by a CMOS camera. Variations in the refractive index of the gaseous samples filling the capillary core could substantially alter the effective refractive index of the core-guided modes, thus shifting the interference fringes. Therefore, sensing of minute changes in the refractive index of the air filling the capillary could be realized by counting the shifted fringes. Moreover, we can also use a photodiode and a slit with a width comparable to that of a single interference stripe (i.e., a bright stripe or a dark stripe) to monitor the amplitude changes in a single-period fringe shift. Experimental results suggest that the sensor proposed has a resolution as high as 5.7 × 10^−8^ RIU, which is comparable to the highest resolution reported in previous literatures [12]. Compared to other fiber-optic gas refractometers utilizing complicated sensing structures or requiring tricky fiber processing steps, the sensor proposed in this paper features the merits of simple structure, straightforward sensing mechanism, ultrahigh sensitivity and resolution, as well as low cost. Virtually any commercial optical capillary could be used as the sensing platform in this sensing scheme, and additional fiber processing—such as fs-laser milling or fusion splicing—is not required. The sensitivity of the sensor depends on the length of the capillary, and thus an even higher sensitivity could be achieved by using a longer capillary. To the best of our knowledge, it is the first time that a gas refractometer is developed based on a single optical capillary. The sensor reported here has strong potential for applications such as ultrasensitive gas refractive index detection and precise air-pressure monitoring. 

## 2. Sensing Mechanism

**Figure 1:**
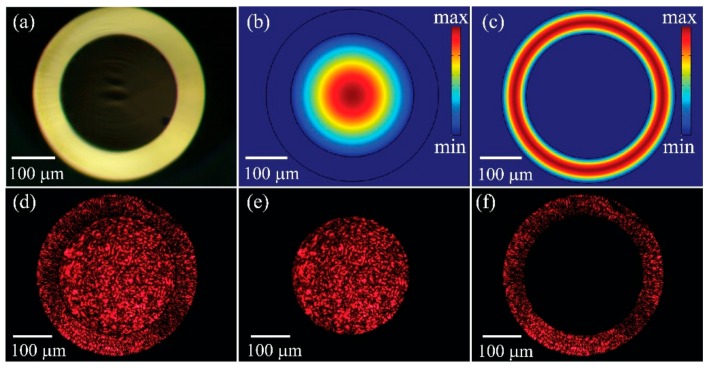
(**a**) Microscopic image of the cross section of the capillary; (**b**) fundamental mode guided by the capillary core; (**c**) fundamental mode guided by the capillary cladding; (**d**–**f**) output of the capillary when HeNe laser beams (λ=632.8 nm) are coupled to (**d**) both of the core and cladding, (**e**) only the hollow core, and (**f**) only the cladding.

The capillary used in our sensor is a commercial product made from borosilicate glass with a refractive index of ~1.51 (Figure 1). The core of the capillary has a diameter of 300 μm and the cladding has a thickness of 60 μm (Figure 1a). Due to the relatively large size of the core and cladding, the in-coupled light thereof would excite a great number of core and cladding modes. A simulation based on finite element method (using COMSOL 5.1) with perfect matched layer (PML) condition is performed to numerically analyze the lowest 50-order modes guided in the core and cladding. In this simulation, we assume that the capillary core is filled with air with a refractive index of 1.0003, and refractive index of the cladding is 1.51 [23,24]. Simulation results indicate that the effective refractive indices of the lowest 50-order core modes and cladding modes are virtually identical to the indices of the air and borosilicate glass, respectively. Besides, the modal crosstalk between the core and cladding modes is negligible. In Figure 1b,c, we only present the modal distribution of the fundamental modes in the core and cladding. We also experimentally visualize the core modes and cladding modes of the capillary by projecting the output light of the capillary to CMOS camera using a 10 × microscopic objective. As shown in Figure 1d, both the core and cladding modes are excited, when two individual laser beams are coupled to the core and cladding, respectively. Note that both the core and cladding modes are highly multimoded, thus leading to a scrambled pattern. By physically blocking the beam coupled to the cladding, only the core modes are left, and vice versa (Figure 1e,f). We can therefore calculate the cross talk between the core modes and cladding modes by comparing the optical power registered in the cladding region to the power in the core region shown in Figure 1e or f. The cross talk is found to be less than −40 dB.

The sensing mechanism of the M-Z capillary interferometer could be simply described as follows. By coupling coherent laser beams to the capillary core and cladding, core modes and cladding modes are simultaneously excited and propagate independently. Variations in the refractive index of gaseous samples filling the core would modify the phase difference φ of the light propagating in the core and cladding as

(1) $$\delta \varphi =k_{0}l\delta n_{core},$$

where $\delta n_{core}$ is the changes in the refractive index of the core; *l* is the length of the capillary; and $k_{0}\;$ is wavenumber defined as $2\pi /\lambda_{0}$, where $\lambda_{0}$ is the operating wavelength in vacuum. Experimentally, transmitted light from the core and cladding would partially overlap and produce interference fringes that would shift in response to variations in the index of the gaseous samples filling the core. A single-period fringe shift accounts for a 2  π variation of φ in Equation (1). The fringe shifts are visualized by a camera. Taking a pixel registering the maximum intensity (grey scale) in a bright stripe as the reference point, we could then count the fringe shifts by counting the number of following shifted bright stripes (maximum grey scales) passing this reference pixel in the camera. An alternative method that could be potentially used for counting the fringe shifts is to apply the fast Fourier transform (FFT) algorithm to process the interferograms (images or videos) to track the phase shifts in the Fourier domain. However, this route is somehow time-consuming, especially when high-frame-rate videos are involved. Thus, it not recommended for real-time monitoring of the fringe shifts.

## 3. Experimental Setup

**Figure 2:**
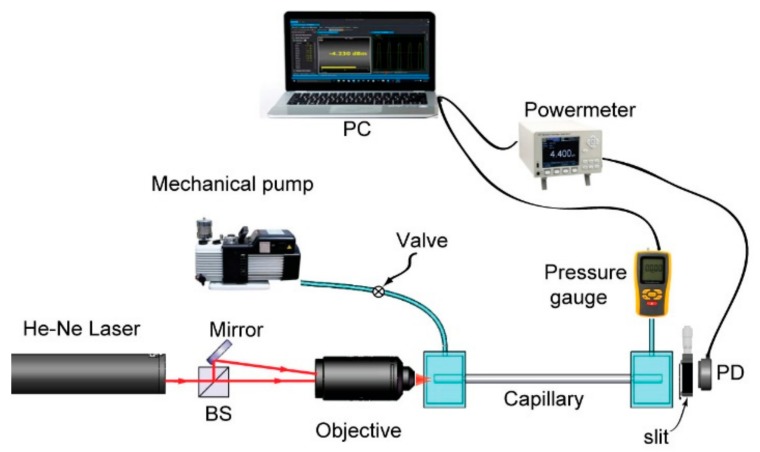
Schematic of the experimental setup of the gas refractometer. BS: beam splitter; PD: photodiode.

Schematic of the proposed capillary sensor is demonstrated in Figure 2. A He-Ne laser (HNL020LB, Thorlabs Inc, Newton, USA) with a wavelength of 632.8 nm and an output power of 1 mW is split into two beams by a cubic beam splitter. Using a 10 × microscopic objective, one beam is coupled directly into the hollow core of a borosilicate capillary (length: 10 cm), while the other beam is firstly reflected by a mirror to propagate virtually parallel to its counterpart and then coupled into the capillary cladding by the same objective. The input end of the capillary sealed in the fluidic block is placed approximately 6.14 mm (working distance of the objective) away behind the objective. The core-guided modes and cladding modes are excited and propagate independently in the capillary waveguide. Here, we would like to note that splitting the laser into two beams for the capillary coupling is not mandatory. By using an objective, one could couple a single laser beam onto the interface between the cladding and the core of a capillary. Accordingly, both cladding modes and core modes could be excited. However, in this route, the laser power attributed to the cladding modes and core modes is sensitive to the coupling condition, which is prone to be affected by surrounding mechanical vibrations. We, therefore, choose to split the laser into two beams and couple the beams onto the cladding and the core, respectively. Due to the relatively large diameter (thickness) of the core and cladding, coupling of the laser beams thereof is facile and less sensitive to environmental vibrations. At the output end, transmitted lights from the core modes and cladding modes interfere, and thus produce an interferogram that is then captured by a CMOS camera. Both ends of the capillary are sealed into the 3D-printed fluidic blocks which enable the air to stream through the capillary rapidly. A transparent window attached on the block enables convenient optical coupling of laser beam into the core and cladding. A mechanical vacuum pump is connected to the block accommodating the capillary input end, and the block holding the other end of the capillary is connected to a digital vacuum gauge with a resolution of 1 hPa (10^2^ Pa). The correlation between the pressure and refractive index of air can be expressed as [12]

(2) $$n_{air}=1+7.82\times 10^{-7}P/\left(273.6+T\right)$$

where *P* is absolute air pressure (Pa), and *T* is temperature (°C). Thus, under a constant temperature, refractive index of the air is linearly proportional to the air pressure. 

## 4. Results and Discussions

**Figure 3:**
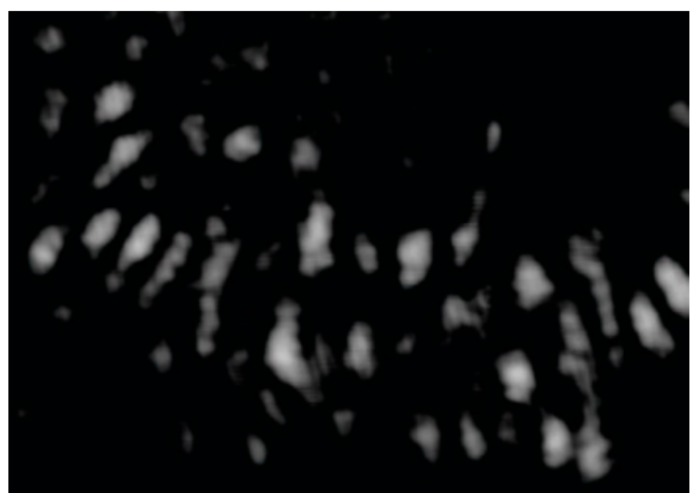
Interferogram partially captured by a CMOS camera at the output end of the capillary, while the inner air pressure of the capillary elevates from 400 to 1012 hPa. Video S1 in the Supplementary Materials.

We first pump out the air in the capillary to lower the internal air pressure to 400 hPa, and then shut down the mechanical pump to let the air back stream slowly till the inner air pressure elevates to surrounding atmosphere pressure (1012.0 hPa). The speed of gaseous samples back-streaming to the capillary could be controlled by a mechanical valve (Figure 2). For the convenience of counting fringe shifts, we experimentally adopted a relatively slow speed. The whole back-streaming period takes ~4 min. During this period, the air pressure inside the capillary is continuously logged by a pressure gauge (VC-9200, Lutron Inc. Dongguan, China) to synchronize with the fringe shift registered by the camera (Figure 3).

**Figure 4:**
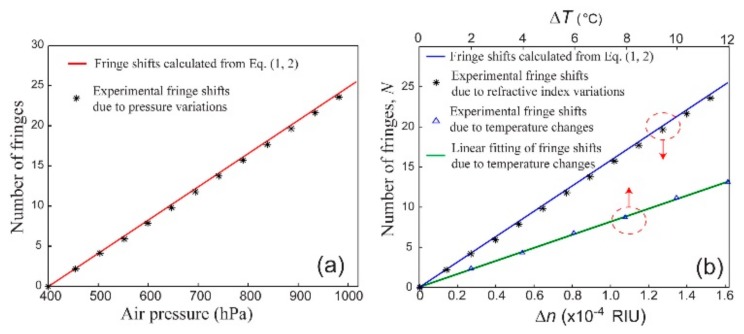
(**a**) Fringe shifts in response to variations in air pressure in the capillary core; (**b**) fringe shifts in response to variations in the refractive index of gas filled in the capillary as well as variations in surrounding temperatures.

We then count the fringe shifts (shown in Video S1 the Supplementary Materials) as a function of air pressure and the corresponding refractive index of the capillary core, respectively, as shown in Figure 4a,b. Thus, the experimental sensitivity of the sensor could be defined as the number of fringe shifts (N=δφ/2π) per refractive index unit (RIU) and is found to be ~1.55 × 10^5^/RIU. Alternatively, we could also define the sensitivity as the variation in the phase difference,  δφ, per refractive index unit, and we found it to be ~9.74 × 10^5^ rad/RIU. Moreover, we also plot the fringe shifts calculated using Equation (1,2) (ambient temperature T = 25 °C). We note that the numbers of experimental fringe shifts, while agreeing well with those in the theoretical prediction, are somewhat smaller than those obtained in the calculation. This may be due to the fact that in our calculation we only consider the change in the refractive index of the gaseous sample filled in the capillary. This change is virtually equivalent to the change in the effective refractive index of the fundamental mode. However, in the practical capillary sensor, many high-order modes could be excited in the core due to the large diameter of the capillary. These high-order modes with smaller fraction of power overlapping with the test analytes (i.e., gaseous samples) filled in the capillary are less sensitive to changes in the refractive index of the analytes as compared to the fundamental mode, thus resulting in a smaller number of fringe shifts [25]. In Figure 4b, the refractive index of the air in the capillary core features an almost perfect linear response to the fringe shifts counted. Thus, assuming a primitive electronic circuit that resolves only a change from the maximum to the minimum of the fringe intensity (0.5 fringe shift) is used, the detection limit of the sensor would be ~3.22 × 10^−6^ RIU. In fact, almost two orders of sensitivity improvement could be gained by analyzing full intensity curves as shown in the following discussion.

In a practical air refractive index sensor, instead of using an expensive CCD or COMS camera we would rather use a single photodiode that samples a small area of the interferogram. In our setup, we use a photodiode detector (S150C, Thorlabs Inc, Newton, USA) that is placed behind an adjustable slit. To ensure the width of the slit is comparable to that of a single bright (or dark) stripe, we first place the camera behind the slit to visualize the interferogram passing through the slit, and then adjust the width and length accordingly. In our sensor, we set the slit with a length of 1 mm and width of ~200 μm. Then, the camera is replaced by the photodiode probe. Once the position of the photodiode and the slit is set, they should be no longer displaced.

**Figure 5:**
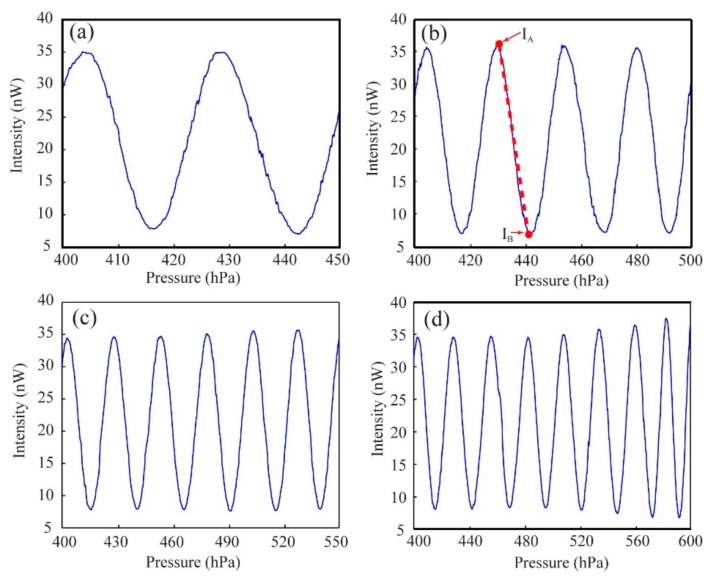
Intensity variations measured by a photodiode detector behind a slit, when the air pressure in the capillary core changes from 400 hPa to (**a**) 450 hPa, (**b**) 500 hPa, (**c**) 550 hPa, and (**d**) 600 hPa.

In Figure 5a–d, we present the recorded intensity variation while the air pressure inside the capillary varies by 50, 100, 150, and 200 hPa. The intensity oscillations in Figure 5 are a direct consequence of the fringe shifts caused by the changes of the air pressure and thus the refractive index of the capillary core. Therefore, the changes of the refractive index could be quantified by counting the number of the periods in the intensity variation. Note that there is also a good repeatability of the sensor. Particularly, the results in Figure 5a–c look simply like cutouts of Figure 5d, although these four measurements are completely independent. 

As mentioned earlier, analysis of intensity variations between two fringes, rather than just counting the number of fringe shifts, may provide a much higher sensitivity. For example, in Figure 5b we could approximately apply a linear fitting between maxima and minima on the second falling edge that corresponds to air pressure changing from 429.8 hPa to 442.2 hPa, and thus a refractive index variation of ~3.24 × 10^−6^ RIU according to Equation (2). Here, the intensity difference between the maximum and the minimum is ~30 nW. To calculate the resolution of the sensor, we need to take the noise level of the sensor into consideration. Thus, we maintain the inner pressure of the capillary constant, and then monitor the long-term peak-to-valley (PTV) intensity fluctuation registered by the photodiode. We experimentally find the PTV fluctuation to be ~1.5% of the registered intensity. Such a fluctuation is attributed to several factors including environmental temperature fluctuations, power fluctuations of the light source, and ambient light noise. To put this noise level into perspective, we could assume the minimal detectable relative change in the optical intensity to be 1.5%, which is ~0.52 nW calculated based on the maximum intensity (~35 nW) in the slope. Thus, the resolution of the sensor can be calculated as ~5.7 × 10^−8^ RIU for gas index sensing. Such a resolution is comparable to the highest resolution that was reported in previous literature [12].

Finally, we would like to comment on the temperature cross sensitivity of the sensor. Perturbations in ambient temperature would lead to changes in the refractive index of the capillary cladding and the air filling the core, as well as minute changes in the diameter and length of capillary, thus resulting in fringe shifts in the interferogram output. The thermo-optic coefficient of borosilicate glass spans a large range depending on the boric content in the glass, so it is challenging to estimate the theoretical temperature cross sensitivity [26]. Experimentally, we investigate the cross sensitivity by heating the capillary sensor using a temperature-controlled breadboard (PTC1, Thorlabs Inc, Newton, USA) under ambient atmosphere pressure, and then counting the fringe shifts as a function of temperature increments. As indicated from the linear fitting in Figure 4b, the cross sensitivity is ~1 fringe shift per degree (°C). Such a cross sensitivity should be considered, when the capillary sensor is used for high-precision measurements especially for a relatively long-term measurement. On the other hand, our capillary sensor offers almost instantaneous response to variations in the gaseous refractive index. In many sensing scenarios that involve quick gas diffusion or pressure variations, changes in the gaseous refractive index occur in a short time window so that influence of thermal perturbations on the sensor could be ignored.

## 5. Conclusions and Future Perspectives

In summary, in this paper we demonstrate a capillary-based M-Z interferometer that could be used for precise detection of variations in refractive indices of gaseous samples. The sensing mechanism is quite straightforward. By coupling coherent beams to the capillary cladding and core, the cladding modes and core modes of a capillary are simultaneously excited. The light transmitted from the core interferes with that transmitted from the cladding, thus producing an interferogram at the output end of the sensor. Changes in refractive index of the capillary hollow core lead to variations in the phase difference between the core modes and cladding modes, thus shifting the interference fringes. We interrogate the fringe shifts using a camera or a photodiode with a narrow slit. The resolution of the sensor is found to be ~5.7 × 10^−8^ RIU that is comparable to the highest resolution obtained by other interference sensors reported in previous literatures. The advantages of our sensor include a very low cost, high sensitivity, straightforward sensing mechanism and ease of fabrication. To further reduce the cost of the sensing system, one might also consider to replace a photodiode detector with a quartz-tuning-fork (QTF) detector, which is normally utilized in the quartz-enhanced photothermal spectroscopy technique or quartz-enhanced photoacoustic spectroscopy technique [27,28]. A QTF detector also features the merits of high-quality factor, small footprint, independent of wavelength, and high responsivity. As the future work of the project, we strive to further lower the cost and optimize the structure of the sensor setup in order to develop a highly cost-effective, compact, portable capillary sensor with an ultra-high sensitivity for gas refractive index detection. Besides, the use of the capillary sensor for detection of refractive index (or concentrations) of specific gaseous sample (e.g., NH_3_, N_2_H_2_) also constitutes an intriguing and promising research direction. One may functionalize the capillary inner or outer surface with certain sensing layers which could selectively absorb the targeted gaseous analytes. Thus, the effective refractive index of the cladding-guided modes would be modified when concentrations of detected gaseous analytes are varied, leading to fringe shifts at the output end of the capillary. While the relevant research is beyond the scope of this paper, we hope that our work would inspire future research endeavors on the development of versatile capillary sensors aiming for detection of a variety of gaseous analytes. 

## Supplementary Materials

The following are available online at https://www.mdpi.com/1424-8220/20/4/1191/s1, Video S1: Fringe shifts Vs air pressure variations.
